# Automated 3D Ultrasound as an Adjunct to Screening Mammography Programs in Dense Breast: Literature Review and Metanalysis

**DOI:** 10.3390/jpm13121683

**Published:** 2023-12-04

**Authors:** Gianluca Gatta, Francesco Somma, Celestino Sardu, Marco De Chiara, Raffaella Massafra, Annarita Fanizzi, Daniele La Forgia, Vincenzo Cuccurullo, Francesco Iovino, Alfredo Clemente, Raffaele Marfella, Graziella Di Grezia

**Affiliations:** 1 Department of Precision Medicine, Università della Campania “Luigi Vanvitelli”, 80131 Napoli, Italy; gianluca.gatta@unicampania.it (G.G.); marc.dechiara93@gmail.com (M.D.C.); alfredo.clemente@unicampania.it (A.C.); 2 U.O.C. Neurodiology, ASL Center Naples 1, P.O. Ospedale del Mare, 80147 Naples, Italy; fra1585@hotmail.it; 3 Department of Advanced Medical and Surgical Sciences, University of Campania Luigi Vanvitelli, Piazza Luigi Miraglia 2, 80138 Naples, Italy; celestino.sardu@unicampania.it (C.S.); raffaele.marfella@unicampania.it (R.M.); 4 Department of Breast Radiology, Giovanni Paolo II/I.R.C.C.S. Cancer Institute, 70124 Bari, Italy; r.massafra@oncologico.bari.it (R.M.); a.fanizzi@oncologico.bari.it (A.F.); d.laforgia@oncologico.bari.it (D.L.F.); 5 Department of Translational Medical Science, School of Medicine, University of Campania Luigi Vanvitelli, 80138 Naples, Italy; francesco.iovino@unicampania.it; 6 Radiology Department, ASL Avellino, 83100 Avellino, Italy; graziella.digrezia@gmail.com

**Keywords:** automatized breast ultrasonography, breast cancer screening, breast

## Abstract

**Purpose**: The purpose of this meta-analysis is to investigate the effectiveness of supplementing screening mammography with three-dimensional automated breast ultrasonography (3D ABUS) in improving breast cancer detection rates in asymptomatic women with dense breasts. **Materials and Methods:** We conducted a thorough review of scientific publications comparing 3D ABUS and mammography. Articles for inclusion were sourced from peer-reviewed journal databases, namely MEDLINE (PubMed) and Scopus, based on an initial screening of their titles and abstracts. To ensure a sufficient sample size for meaningful analysis, only studies evaluating a minimum of 20 patients were retained. Eligibility for evaluation was further limited to articles written in English. Additionally, selected studies were required to have participants aged 18 or above at the time of the study. We analyzed 25 studies published between 2000 and 2021, which included a total of 31,549 women with dense breasts. Among these women, 229 underwent mammography alone, while 347 underwent mammography in combination with 3D ABUS. The average age of the women was 50.86 years (±10 years standard deviation), with a range of 40–56 years. In our efforts to address and reduce bias, we applied a range of statistical analyses. These included assessing study variation through heterogeneity assessment, accounting for potential study variability using a random-effects model, exploring sources of bias via meta-regression analysis, and checking for publication bias through funnel plots and the Egger test. These methods ensured the reliability of our study findings. **Results:** According to the 25 studies included in this metanalysis, out of the total number of women, 27,495 were diagnosed with breast cancer. Of these, 211 were diagnosed through mammography alone, while an additional 329 women were diagnosed through the combination of full-field digital mammography (FFDSM) and 3D ABUS. This represents an increase of 51.5%. The rate of cancers detected per 1000 women screened was 23.25‰ (95% confidence interval [CI]: 21.20, 25.60; *p* < 0.001) with mammography alone. In contrast, the addition of 3D ABUS to mammography increased the number of tumors detected to 20.95‰ (95% confidence interval [CI]: 18.50, 23; *p* < 0.001) per 1000 women screened. **Discussion:** Even though variability in study results, lack of long-term outcomes, and selection bias may be present, this systematic review and meta-analysis confirms that supplementing mammography with 3D ABUS increases the accuracy of breast cancer detection in women with ACR3 to ACR4 breasts. Our findings suggest that the combination of mammography and 3D ABUS should be considered for screening women with dense breasts. **Conclusions:** Our research confirms that adding 3D automated breast ultrasound to mammography-only screening in patients with dense breasts (ACR3 and ACR4) significantly (*p* < 0.05) increases the cancer detection rate.

## 1. Introduction

Rationale

Breast cancer was the most diagnosed cancer in 2020, accounting for 2.3 million cases (11.7% of all new diagnoses), followed by lung cancer (11.4%), colorectal cancer (10%), prostate cancer (7.3%), and stomach cancer (5.6%). Regarding mortality, lung cancer topped the list, causing 18% of all cancer-related deaths, followed by colorectal and liver cancers. The International Agency for Research on Cancer estimated that Europe sees 464,000 new breast cancer cases annually, with 99% of them occurring in women. In Italy, breast cancer took the lead among all cancer types in 2020, with around 55,000 cases. It was the most prevalent cancer in women across all age groups, impacting the population differently based on age (41% in those under 49 years vs. 22% in older individuals). The 5-year relative survival rate in Italy stood at 87%, surpassing the European average of 82%.

In the United States, there has been a 30% increase in breast cancer cases over the past five years, with an annual incidence of 281,550 new cases. Despite remaining the most common cancer in women, screening and early diagnosis interventions have led to a consistent reduction in mortality rates among all major ethnic groups [1]. Full Field Digital Mammography (FFDM) serves as a potent screening tool for early breast cancer detection, but it is not without flaws, especially in women with dense breasts. Women with dense breasts face a higher breast cancer risk and mammography exhibits reduced sensitivity across all age groups. The use of 3D Automatized Breast Ultrasonography (3D ABUS) has proven effective in detecting mammographically occult cancer in women with dense breast tissue. Studies have demonstrated that 3D ABUS significantly enhances the detection of clinically significant, small, largely invasive, node-negative cancers.

Overall, the sensitivity of mammography in breast cancer detection is approximately 85%. However, this sensitivity drops to 47.8% to 64.4% when dealing with women with dense breast tissue. Furthermore, women with extremely dense breasts are 4.7 times more likely to develop breast cancer. While 30 U.S. states have “density notification” laws requiring women to be informed about their breast density, in Italy, it is the responsibility of the doctor to provide this information. Nevertheless, there remains a need to increase awareness among the patient population.

Objectives

The primary objective of this article is to evaluate the influence of these screening methodologies on cancer detection rates, with specific attention given to high-risk and intermediate-risk women. This article highlights the importance of confronting the complexities associated with breast cancer screening, particularly among women with dense breast tissue, and accentuates the potential advantages of integrating 3D ABUS into the screening protocol.

## 2. Materials and Methods

### 2.1. Methodology

The methodology employed in this scientific paper aligns with the guidelines established by the PRISMA (Preferred Reporting Items for Systematic Reviews and Meta-Analyses) statement, ensuring the comprehensive and transparent reporting of our systematic review [2]. Our rigorous adherence to these guidelines exemplifies our commitment to conducting a thorough and well-documented review. Furthermore, we diligently followed the PROSPERO guidelines for registration. However, it is worth noting that PROSPERO typically does not accept submissions for scoping reviews, literature reviews, or mapping reviews. Consistent with their recommendations, we submitted both the complete protocol and the finished review, adhering to the highest standards of research transparency.

### 2.2. Research Strategies

#### Information Sources

In November 2021, we executed a comprehensive bibliographic search in the MEDLINE (PubMed) and Scopus databases. This search was carried out by two experienced researchers with a deep understanding of breast cancer pathology. Our search string was as follows: (‘3D abus’ /exp OR ‘Abus/exp’ OR ‘mammography’/exp OR ‘breast cancer’/exp) AND (‘screening’/exp OR ‘3D Abus’) AND (‘intraductal carcinoma’/exp OR ‘intraductal carcinoma of the breast’ OR ‘breast carcinoma’ OR ‘ductal carcinoma’ OR ‘intraductal carcinoma’ OR ‘breast cancer’/exp OR ‘carcinoma, mammary’ OR ‘invasive ductal carcinoma’ OR ‘mammary carcinoma’) AND ‘article’/it.

### 2.3. Eligibility Criteria

#### 2.3.1. Inclusion Criteria

Our analysis focused exclusively on original articles pertaining to human patients. These articles were required to be written in English and published between 2000 and 2021. Additionally, only articles with available abstracts were included for examination. This stringent set of criteria was designed to ensure that our study remained consistent by selecting articles that met specific requirements. This rigorous approach allowed us to conduct a standardized and thorough analysis.

#### 2.3.2. Selection Process

Articles were initially chosen based on their title and abstract. The number of patients evaluated in the selected articles was restricted to a minimum of 20. Any articles that did not meet these criteria were promptly excluded from consideration. Eligible articles were then downloaded, and the same two readers who conducted the initial selection independently assessed the full text.

#### 2.3.3. Outcome

**Figure 1:**
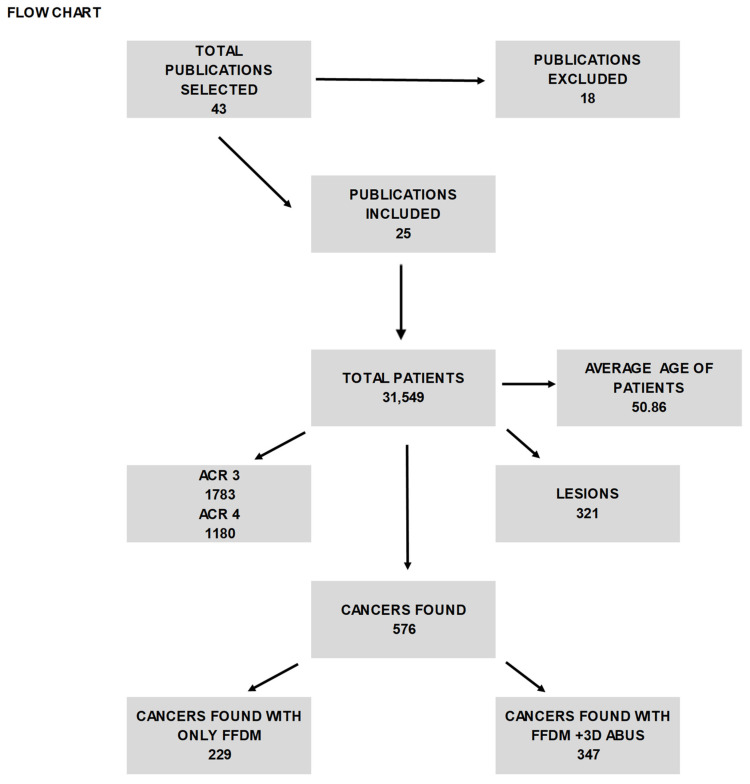
The table shows the selected articles from 2000 and 2021 and the features of patients and lesions found with FFDM and ABUS.

**Figure 2:**
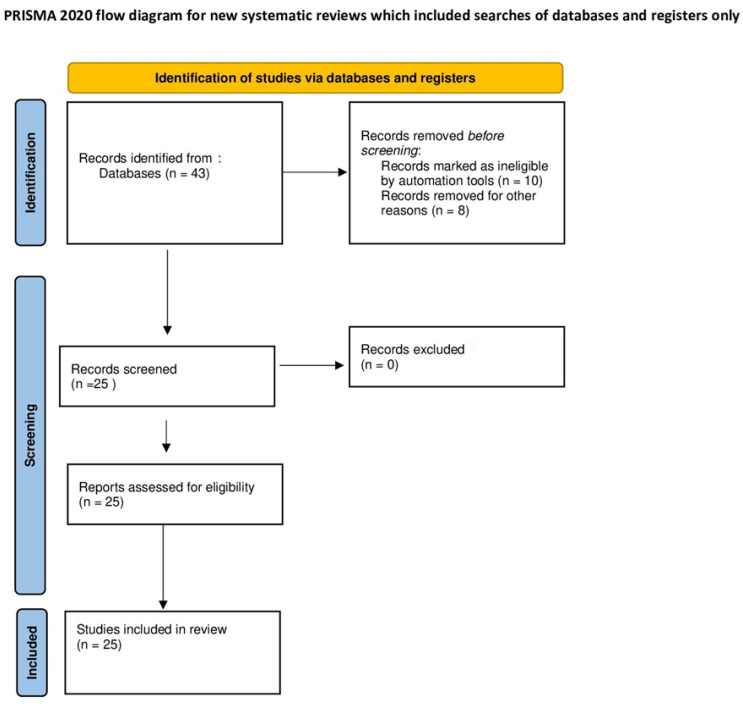
Flow diagram of the studies identified for the meta-analysis.

The search initially generated 43 articles (as illustrated in Figure 1), comprising 17 from PubMed and 26 from Scopus. After a careful review, articles that were deemed unsuitable were removed, resulting in a total of 25 articles that met our inclusion criteria (as depicted in Figure 2 and summarized in Table 1). The selection process and the number of articles included are illustrated in the accompanying figure.

### 2.4. Selection of Articles

#### Search Strategy and Selection Process

Our search strategy and selection process involved a meticulous evaluation of numerous abstracts, guided by specific criteria to ensure the inclusion of high-quality articles. The chosen studies were required to be published in peer-reviewed scientific journals and composed in English, signifying a commitment to scientific rigor and quality. Furthermore, these studies needed to involve participants aged 18 or older from select geographical regions, including the United States, Canada, Europe, Australia/New Zealand, China, Japan, or Egypt. Quantitative measures and a minimum sample size of more than *n* = 20 were also essential criteria. The use of peer-reviewed scientific journals guaranteed that the selected articles had undergone rigorous evaluation by experts in the field and met the established scientific standards.

Regrettably, for practical reasons, studies not published in English were omitted from our selection process, as this would have impeded our ability to conduct a comprehensive review. During the initial screening, 18 items were eliminated based on these criteria, leaving us with a subset of articles to be evaluated in their entirety by the authors.

In our comprehensive assessment of these articles, we considered various factors as potential grounds for exclusion. Articles were excluded if they failed to provide relevant correlations, lacked quantitative measurements, or did not assess any psychological or physical variables. In many instances, articles were deemed unsuitable due to the absence of essential inclusion criteria, while others were discarded due to a combination of multiple criteria that were not met.

This rigorous search strategy and selection process ensured that the articles included in our study were of the highest quality and relevance, meeting our stringent criteria for robust scientific investigation.

### 2.5. Data Extraction

#### 2.5.1. Data Collection Process and Data Items

For each article analyzed, the recorded data encompassed various aspects such as the year of publication, study design, number of patients and lesions, average patient age, the prevalence of personal and/or family history of breast cancer among patients, identified lesions, mean lesion size observed during imaging, type of biopsy conducted (core needle biopsy, vacuum-assisted biopsy), patients with ACR3 and ACR4, patients with BI-RADS 3-4-5, identified cancers (FFDSM and FFDSM and 3D ABUS only), recall rate ‰ (FFDSM and FFDSM and 3D ABUS only), cancer detection ‰ (FFDSM and FFDSM and 3D ABUS only), sensitivity% (FFDSM and FFDSM and 3D ABUS only), and specificity (FFDSM and FFDSM and 3D ABUS only).

#### 2.5.2. Study Risk of Bias Assessment, Reporting Bias Assessment, and Certainty Assessment

In our study, we employed a rigorous approach to assessing risk of bias, reporting bias, and the overall certainty of the findings.

To evaluate the potential bias in our study, it is important to note that we did not account for multiple lesions within individual patients. This limitation was acknowledged, and we carefully considered its implications. We conducted a critical appraisal of the study design, data collection, and analysis methods to identify potential sources of bias and addressed them to the best of our ability.

In order to minimize reporting bias, we collected data from the same two independent readers who were responsible for conducting the literature search and selecting relevant studies. This approach helped ensure consistency and reduce the risk of selective reporting or data manipulation. Any discrepancies in the data were thoroughly reviewed and resolved by a third reader with extensive experience in breast imaging spanning over 20 years. This expert input further enhanced the accuracy and completeness of our data.

While our study diligently followed established research standards and procedures, we acknowledge that every study has inherent limitations. The certainty of our findings was assessed by considering the overall quality and reliability of the data, the study design, and potential sources of bias. Our use of experienced readers and experts in breast imaging helped enhance the confidence in our results. By carefully addressing potential bias, maintaining consistency in data collection, and involving a seasoned expert in breast imaging, we aimed to uphold the integrity and reliability of our study’s findings. These measures were taken to provide a thorough and transparent assessment of breast cancer screening, acknowledging the study’s limitations and uncertainties (Table 2).

### 2.6. Endpoints

Primary Endpoint: Our primary objective is to delve into the effectiveness of augmenting traditional screening mammography with the three-dimensional automated breast ultrasonography. We aim to assess the impact of this combined screening approach on enhancing breast cancer detection rates among asymptomatic women who present the challenge of dense breast tissue. In doing so, we aspire to offer valuable insights into the potential improvements that can be achieved in early breast cancer detection, a key factor in improving patient outcomes and reducing the burden of this disease.

Secondary Endpoint: In addition to our primary focus, we also have a secondary endpoint in our study. We intend to examine and compare the performance of two different breast cancer screening modalities, namely, full-field digital mammography and three-dimensional automated breast ultrasonography. By investigating the distinctions between these screening methods, we aim to elucidate the nuances in screening efficacy, detection outcomes, and diagnostic accuracy. Such insights into the comparative strengths and limitations of these technologies can contribute to informed decision-making in breast cancer screening protocols.

### 2.7. Statistical Analysis

#### Synthesis Methods

In our study, we implemented a comprehensive set of statistical techniques using MedCalc^®^ Statistical Software version 20.011 (MedCalc Software Ltd., Ostend, Belgium; https://www.medcalc.org, accessed on 28 September 2022) for statistical analysis to pinpoint and mitigate potential biases.

Through heterogeneity analysis, we scrutinized the variability among the chosen studies, aiming to comprehend the differences between them. High heterogeneity, signified by an I2 exceeding 50%, could signify potential bias sources. The DerSimonian Random-Effects Model T and Laird method were employed. This strategy accounts for possible study-to-study variations and aids in bias reduction while determining the pooled effect size. The reference standards for the analysis included histopathologic assessment following surgical excision and follow-up imaging for lesions that did not undergo surgical excision [27]. Subgroup analyses were also performed. Each potential covariate was individually considered in the meta-regression analysis, excluding the intercept. The goodness of fit for each model was assessed using R2 analog statistics and tests for residual variance. The aim was to include the minimum number of covariates that maximized the R2 value and yielded a nonsignificant test of unexplained variance [28]. We conducted meta-regression analysis to investigate potential bias and variation sources and to account for the observed heterogeneity. This entailed evaluating individual covariates to discern their impact on study outcomes [29,30]. To identify potential publication bias, we made use of funnel plots and conducted the Egger test. Funnel plots were visually assessed for data distribution symmetry, while the Egger test furnished a quantitative gauge of potential bias. These statistical methodologies played a crucial role in both detecting potential bias origins and diminishing their influence on our study’s outcomes. This ensured the dependability and validity of our findings.

## 3. Results

### 3.1. Study Selection

This study encompassed 43 publications and a total of 31,549 patients, with an average age of 50.86 years. However, following meticulous scrutiny, only 25 of these scientific papers, accounting for 27,495 patients, conformed to the study’s criteria.

### 3.2. Study Characteristics

In the comprehensive cohort of patients considered in our study, we observed a diverse range of characteristics and diagnostic outcomes, reflecting the rich complexity of breast cancer screening. Among the 27,495 patients included, there were 1754 cases classified as ACR3 and 1152 cases classified as ACR4, indicative of varying levels of breast density. Additionally, our data encompassed 12,253 patients with BI-RADS (Breast Imaging Reporting and Data System) classification, 3,3943 patients with BI-RADS 4, and 260 patients with BI-RADS 5, underscoring the broad spectrum of breast imaging findings encountered in clinical practice.

Across the compendium of publications we scrutinized, a total of 211 cases of breast cancer were identified through Full Field Digital Mammography (FFDSM) alone, while the combination of FFDSM and 3D Automated Breast Ultrasound System (ABUS) led to the detection of 329 cases. Notably, the average lesion size, measured in millimeters, was 14.86 for FFDSM alone and slightly smaller at 13.62 when FFDSM was complemented by 3D ABUS.

Interestingly, when evaluating the recall rate, we noted a distinctive pattern. The utilization of FFDSM alone was associated with a recall rate of −1.28 per thousand patients, suggesting that fewer patients were called back for further evaluation than expected. However, this rate increased significantly to 11.89 per thousand patients when both 3D ABUS and FFDSM were employed, indicating a higher level of vigilance in follow-up assessments.

As we delved deeper into the assessment of cancer detection rates, we encountered varying findings among the articles under consideration. One source, denoted as [8], proposed that the cancer detection rate is comparable for 3D ABUS and FFDSM. Nevertheless, other studies examined in this context presented contrasting perspectives. On average, the cancer detection rate was calculated at 28.1 per thousand patients for FFDSM alone, whereas it amounted to 17.4 per thousand patients for 3D ABUS. However, it is crucial to acknowledge the influence of individual studies on this aspect. When we excluded the data from source [8], the detection rate exhibited a notable shift, and the preference for cancer detection methods changed accordingly. In this scenario, FFDSM alone appeared to detect 4.3 per thousand cases of breast cancer, while the combination of 3D ABUS and FFDSM identified 6.6 per thousand cases of breast cancer, indicating an adjustment in the comparative efficacy of these screening modalities.

The diversity among the articles included in our study becomes even more pronounced when examining sensitivity and specificity. The overall sensitivity for FFDSM alone was calculated at 65.1%, a value that significantly increased to 82.7% when FFDSM was augmented with 3D ABUS. However, it is important to note that individual studies, conducted by various authors in different geographical regions, yielded differing results. This underscores the multifaceted nature of breast cancer screening, with varying outcomes driven by diverse patient populations, healthcare practices, and research methodologies.

### 3.3. Results of Individual Studies

The landscape of breast cancer screening is rife with complexity, as evidenced by the diverse findings across various studies. Park et al. [17] reported that FFDSM alone exhibited a sensitivity of 44.7%, which notably dropped to 22.2% when FFDSM was combined with 3D ABUS. In stark contrast, Brem et al. presented a distinct perspective, indicating that the combination of both methods propelled sensitivity from an already commendable 73.2% with FFDSM alone to a remarkable 100% when 3D ABUS was integrated. The spectrum of results is further broadened by other studies, which fall somewhere between these extremes, yielding sensitivity values that fluctuate based on the specific study in question. These variations highlight the multifaceted nature of breast cancer screening and the myriad factors that influence its outcomes.

On the front of specificity, there is a greater consensus among authors that the combination of 3D ABUS and FFDSM often leads to a reduction in specificity levels. The average specificity experienced a marginal decline, shifting from 93.3% to 90.1% when both methods were employed concurrently. Nevertheless, Giuliano et al. [5] and Kelly et al. [22] notably bucked this trend, observing an increase in specificity when 3D ultrasound was paired with FFDSM. Their studies demonstrated specificity values ascending from 98.2% to 99.7% and from 95.1% to 98.7%, respectively, highlighting the diversity of findings even in this aspect of breast cancer screening. The application of automated whole breast ultrasound (ABUS) in cases of dense breast tissue results in a remarkable boost in sensitivity for breast cancer detection. This enhancement is particularly pronounced when it comes to identifying small or invasive cancers, with denser breast tissue exhibiting a more substantial increase in sensitivity compared to less dense breast tissue. Nevertheless, it is important to note that this sensitivity gain might come at the cost of a slight reduction in specificity [22].

The significance of breast density cannot be overstated in the context of breast cancer screening. Breasts characterized by a high proportion of glandular tissue are referred to as dense breasts. They pose a dual challenge: firstly, they can render mammograms more challenging to interpret, potentially obscuring early signs of cancer. Secondly, they elevate the risk of developing tumors by up to six-fold, underscoring the seriousness of this condition. Alarmingly, this condition affects approximately half of all women over the age of 40, emphasizing the critical need for heightened awareness and proactive healthcare measures.

**Figure 3:**
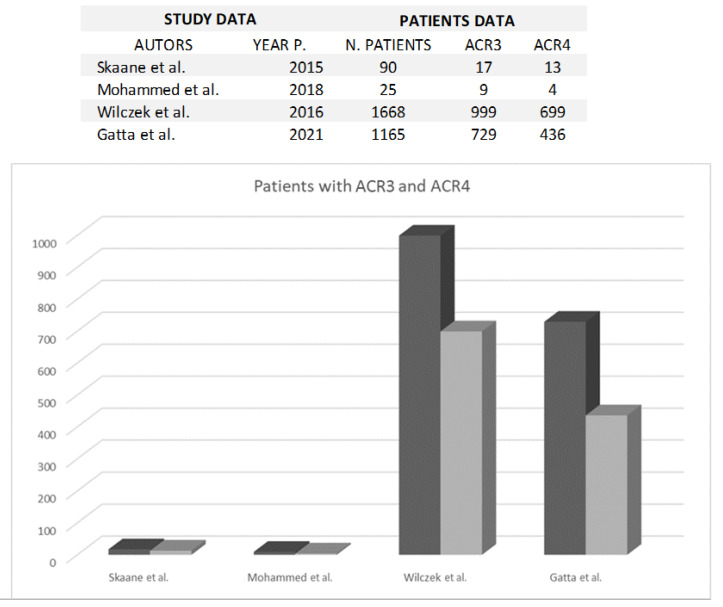
The figure shows the comparison between ACR 3 (black) and ACR 4 (grey) breast density classification in each study [3,8,11,26].

The American College of Radiology (ACR) system categorizes women into four groups based on the amount of glandular tissue visible on mammograms. Women categorized as “C” and “D,” termed “heterogeneously dense” and “extremely dense,” respectively, face a significantly heightened risk of breast cancer. Two specific studies delved into the incidence of these categories, encompassing 729 and 999 patients in Category “C,” and 436 and 699 patients in Category “D,” as outlined in Figure 3.

This multifaceted interplay of sensitivity, specificity, and breast density underscores the intricate nature of breast cancer screening, demonstrating the need for personalized approaches and tailored strategies to address the diverse challenges encountered in clinical practice.

In the context of our study, it is pertinent to understand the implications of different BI-RADS levels. BI-RADS 3, as observed across various studies, typically signifies a probable benign finding, often warranting a recommendation for short-term follow-up to monitor any potential changes. On the other hand, BI-RADS 4 introduces a spectrum of possibilities, encompassing low, intermediate, and high suspicion of malignancy. This categorization prompts a more nuanced and detailed evaluation of breast abnormalities to determine the level of risk and guide further action. Finally, BI-RADS 5 represents a conclusive finding of malignancy, signaling the necessity for immediate and decisive medical intervention.

**Figure 4:**
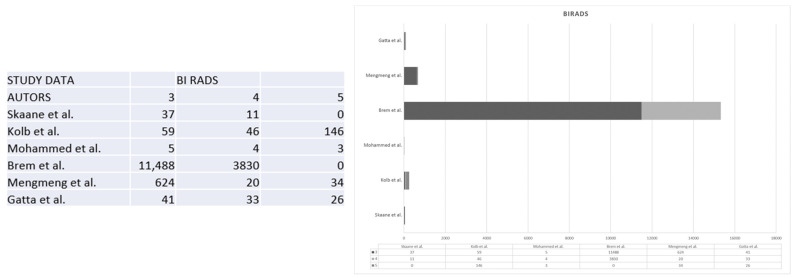
The figure shows the comparison between the BIRADS classification in 3, 4, and 5 of the lesions in the different studies [3,7,8,18,21,26].

It is worth noting that the prevalence of BI-RADS 5 findings can vary across different studies, reflecting the diversity of patient populations and healthcare practices. For instance, Kolb et al. reported a higher occurrence of BI-RADS 5 findings compared to studies conducted by Brem et al., Mengmeng et al., and Gatta et al [18,21,26]. This variation is often proportional to the number of participants enrolled in these studies, as outlined in Figure 4 [3,7,8,18,21,26]. Second-generation automated breast ultrasonography (ABUS) represents a substantial leap forward when compared to first-generation ABUS. The technology integrated into this generation has undergone significant refinement, resulting in sharper, clearer, and more detailed images. These improvements facilitate a more accurate interpretation of the ultrasound scans. In addition to superior image quality, second-generation ABUS also boasts an expanded coverage of breast tissue. The ultrasound scans generated by this technology encompass a broader area of the breast, leaving fewer regions unexamined. This comprehensive coverage increases the likelihood of detecting breast abnormalities, even in the most challenging cases, such as those involving women with dense breast tissue. Furthermore, the second generation of ABUS incorporates advanced image analysis algorithms designed to assist radiologists in the interpretation of ultrasound scans by identifying potential abnormalities and highlighting areas of concern. By doing so, they not only aid in improving the accuracy of detection but also contribute to a reduction in false-negative results. Reducing false negatives is a pivotal aspect of breast cancer screening, as it directly impacts patient outcomes. In the context of second-generation ABUS, the advancements in technology and image analysis translate into a notable reduction in cases where breast abnormalities are missed during screening. This means that more instances of breast cancer can be identified at an earlier, more treatable stage, which, in turn, contributes to improved patient outcomes [26].

### 3.4. Results of Syntheses and Reporting Biases

Analysis of the referenced studies reveals a range of critical considerations regarding potential biases that could influence the outcomes of this meta-analysis. Firstly, the process of selecting publications, which initially included 43 and was later narrowed to 25, brings forth concerns about selection bias. The criteria used for study inclusion might inadvertently favor specific research types, possibly influencing the overall findings. Heterogeneity among the selected studies, as indicated by I2 values, suggests the presence of various potential sources of bias. These disparities may arise from differences in study design, the characteristics of study populations, and other factors that impact the comparability of the studies. While funnel plots and the Egger test are valuable tools for detecting publication bias, it is crucial to acknowledge that publication bias may persist despite these measures. Variability in sensitivity and specificity across studies conducted by different authors suggests the potential for bias. This variation can be attributed to differences in research methodology, patient populations, data collection techniques, and interpretation standards. Selection bias may also play a role, as researchers may choose to include or exclude specific data or participants in ways that do not fully represent the broader population. These choices may not always be adequately documented in research papers, potentially leading to biased outcomes. Sample size differences among studies can contribute to result discrepancies, with smaller studies often exhibiting more variability and potentially producing biased estimates of sensitivity and specificity.

Furthermore, variations in data collection methods, patient populations, and interpretation standards can lead to inconsistencies in findings. The presentation of odds ratios indicating both positive and negative associations with the disease raises questions about the consistency of risk factor assessments across studies. Variability in study design, sample sizes, or adjustments for covariates may contribute to this unpredictability. The range of results regarding the impact of 3D ABUS on cancer detection rates points to potential biases arising from differences in patient selection, equipment quality, and interpretation methodologies. The influence of breast density on mammography results introduces concerns about potential bias in radiologist interpretations, as breast density significantly affects the detection of abnormalities. Variations in the prevalence of ACR and BI-RADS classifications across studies may be linked to differences in interpretation standards, potentially introducing bias.

In summary, these intricacies highlight the need for a careful and cautious interpretation of the meta-analysis results, considering the potential biases and sources of heterogeneity that underlie the findings.

**Figure 5:**
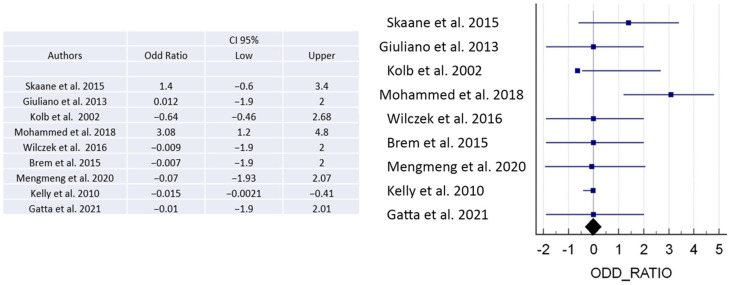
The figure shows the odds ratio to demonstrate positive or negative association with the disease [3,5,7,8,11,18,21,22,26].

There is a clear link between particular risk factors and the onset of a disease, as evidenced by a numerical ratio. A ratio of less than 1 denotes a negative correlation, indicating a protective effect against the disease. Conversely, a ratio higher than 1 suggests a positive correlation, meaning a higher likelihood of developing the disease. The magnitude of the association becomes stronger with greater distance from a ratio of 1 in either direction. When examining the odds ratio from different publications, we can identify a likelihood ratio among the data of some of the studies. While some studies show odds ratios greater than 1 indicating a positive association with the disease, others demonstrate an odds ratio of less than 1, indicating a negative association [3,7,8,11,18,21,26] (Figure 5).

**Figure 6:**
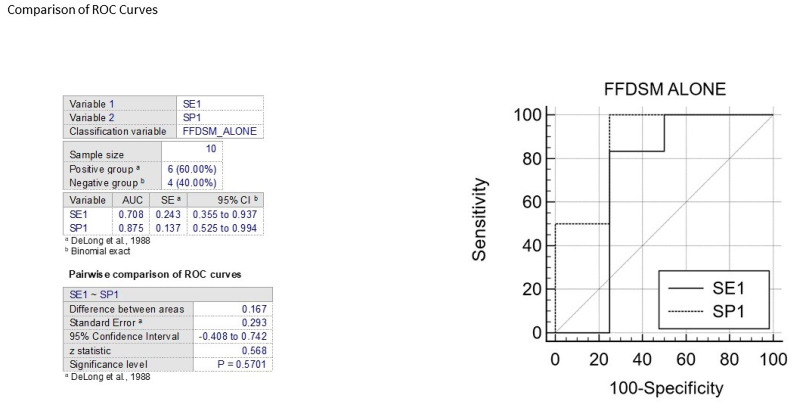
The figure shows the comparison between ROC curves to analyze sensitivity and specificity of FFDM alone [32].

**Figure 7:**
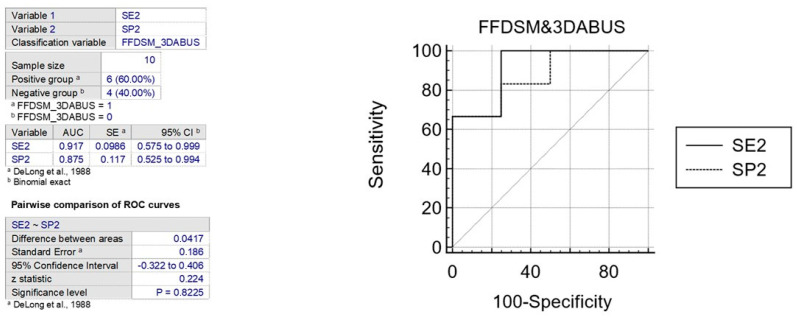
The figure shows the comparison between ROC curves to analyze sensitivity and specificity of FFDM and B3D AUS [32].

The ROC curve is a statistical tool that measures the accuracy of a diagnostic test across a range of possible values. It is the preferred method for validating diagnostic tests and evaluating the specificity and sensitivity of mammography alone or in combination with 3D ABUS. The ROC curve also allows for the identification of the optimal threshold value using the Youden index [31]. To interpret the AUC value, which ranges from 0.5 (not informative) to 1 (perfect test), reference values are used. A test is considered more accurate if its ROC curve is closer to the upper left angle of the graph. The cut-off value that maximizes both sensitivity and specificity is represented by the point closest to this angle. (Figure 6 and Figure 7).

**Figure 8:**
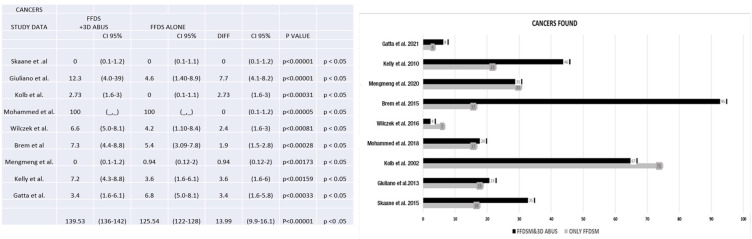
The figure shows the number of cancers found with FFDM alone and FFDM and 3D ABUS [3,5,7,8,11,18,21,22,26].

In various publications, the specificity and sensitivity of mammography alone have been evaluated using the ROC curve, resulting in AUC values between 0.70 and 0.87 for two variables. The comparison of two ROC curves showed an area difference of 0.167% and 0.041%, respectively. Based on these findings, the first graph indicates that FFDSM alone is moderately accurate, while the second graph indicates that FFDSM and 3D ABUS is highly accurate (Figure 8).

### 3.5. Certainty of Evidence

Regarding the identification of cancers, we conducted an analysis of nine publications [3,5,7,11,18,19,21,22,26]. Our examination of these studies yielded a diverse spectrum of results. Notably, our findings consistently demonstrated that the integration of 3D Automated Breast Ultrasound alongside full-field digital mammography consistently resulted in an increased frequency of cancer detection. This observation underscores the potential value of combining these two screening modalities in enhancing the early detection of breast cancer.

## 4. Discussion

Breast cancer remains a significant global health challenge, characterized by high incidence and mortality rates. It is imperative to employ effective screening methods not only to save lives but also to enhance the quality of life for those affected by this common disease [33,34]. Mammography, the primary tool for breast cancer screening, is widely available, cheap, efficient, and does not require advanced training to be used. However, it has inherent limitations, particularly in women with dense breast tissue [35,36]. Breast density plays a pivotal role in radiological assessment, significantly impacting breast cancer screening and diagnosis and, therefore, the survival outcome of our patients [37]. Breast density categorizes breasts into four groups: fatty, scattered fibroglandular density, heterogeneously dense, and extremely dense. Dense breasts have a higher proportion of fibroglandular tissue, while fatty breasts contain more fatty tissue [4,6]. The issue of breast density primarily affects mammography, the most commonly used breast cancer screening method. The overlap in density between glandular tissue and potential tumors can obscure small abnormalities, making them challenging to detect. This may lead to reduced sensitivity, particularly in women with dense breasts [9,10,12].

Although alternative advanced imaging techniques such as MRI and nuclear medicine procedures (PET and lymphoscintigraphy) are crucial for cancer staging and surgery, they are not currently the primary diagnostic methods [38,39]. MRI stands out for its safety during pregnancy, excellent spatial and contrast resolution, absence of ionizing radiation, and non-operator-dependent results [40].

This article aims to consolidate existing literature on breast cancer screening, exploring the advantages of using mammography as a standalone method and the potential benefits of augmenting it with 3D automated breast ultrasound (ABUS), especially for high-risk and intermediate-risk women. The value of 3D ABUS lies in its substantial potential to enhance breast cancer detection rates, particularly in women with dense breast tissue [21]. It functions as a complementary approach to mammography, offering supplementary imaging information. Automated ultrasound technology is employed to create a three-dimensional representation of the breast, enabling a more comprehensive and detailed evaluation of breast tissue [12]. Numerous studies underscore 3D ABUS’s ability to offer a more thorough assessment of breast tissue, capable of revealing abnormalities that might remain concealed on mammograms. This, in turn, facilitates earlier detection and potentially improved treatment outcomes, addressing the limitations of mammography, especially in women with dense breasts [3,5,7,8,17,18,21,22,26].

Several studies demonstrate that 3D ABUS significantly elevates the detection of clinically relevant breast cancers, especially small, invasive, and node-negative ones, which might be missed by mammography alone [13]. Considering the articles reviewed, which focus on breast cancer screening and compare the detection outcomes of mammography alone to its combination with 3D ABUS, the additional value of 3D ABUS becomes evident [14,15,27]. A comprehensive analysis of these studies consistently reveals that integrating 3D ABUS with mammography leads to heightened sensitivity and improved cancer detection rates compared to mammography alone [16]. While variations may exist in the results of different studies, on average, the combination of 3D ABUS and mammography enhances accuracy compared to mammography alone [41].

Importantly, the incorporation of 3D ABUS with mammography has the potential to reduce false-negative results and unnecessary recalls, which can result in cost savings [20]. By enhancing the accuracy of breast cancer screening, 3D ABUS can contribute to better patient outcomes by enabling early detection and timely intervention. It is crucial to emphasize that 3D ABUS typically serves as an adjunctive screening tool and is not intended to replace mammography [23]. Mammography remains the cornerstone of breast cancer screening, and the addition of 3D ABUS complements the overall sensitivity and precision of this process, especially for women with dense breast tissue [25,42]. The decision to incorporate 3D ABUS into breast cancer screening protocols should be individualized, based on patient considerations and clinical judgment.

Regarding the review process, it is noteworthy that the review was not registered, and no review protocol was developed. As such, there have been no amendments or changes to registration or protocol-related information. The authors conducted the review independently, without involvement or influence from funders or sponsors in the review’s design, execution, or reporting. Concerning data and materials availability, none of these are publicly accessible.

## 5. Conclusions

Our meta-analysis stands as an exhaustive and rigorous investigation, delving into a multitude of studies, each meticulously examining the effectiveness of mammography in isolation and its integration with 3D Automated Breast Ultrasound (ABUS) for the early detection of breast cancer. This extensive synthesis of data drawn from a diverse array of publications unravels a compelling narrative that underscores the substantial advantages inherent in adopting a combined approach that employs both mammography and 3D ABUS. This approach not only significantly enhances the detection of breast cancer but also provides a consistent and noteworthy pattern of substantial improvements in sensitivity.

The central theme that emerges from our comprehensive analysis is the undeniable enhancement in sensitivity achieved by amalgamating these screening methods, even if it necessitates a slight trade-off in specificity. This pivotal discovery serves as a resounding endorsement for the inclusion of both mammography and 3D ABUS within the framework of mammographic screening protocols. This endorsement holds particular weight when considering women with dense breast tissue, who stand to benefit greatly from this integrated approach.

However, it is essential to recognize that the role of 3D ABUS in breast cancer screening is continually evolving. Ongoing research and clinical assessments are poised to further fine-tune its position and utility in the broader landscape of healthcare. As we navigate this dynamic landscape of medical advancements, the integration of 3D ABUS holds the promise of making a substantial impact. It contributes to the early detection of breast cancer, a crucial factor in improving patient outcomes and, ultimately, in the noble mission of saving lives.

## Figures and Tables

**Table 1:** The table shows the 25 selected articles, the year of the article, patient’s data, number of lesions, BIRADS, the cancer found, the recall rate, the cancer detection rate, and the diagnosis with FFDM and ABUS.

STUDY DATA							BI RADS			Cancers Found			Size Cancer mm			Recall Rate (‰)		Cancer Detection Rate (‰)		SE		SP	
AUTORS	YEAR	N. Patients	Age	ACR3	ACR4	LESIONS	3	4	5	Only FFDSM	FFDSM + 3D ABUS	TOT	Only FFDSM	FFDSM + 3D ABUS	DIFF	FFDSM + 3D ABUS	Only FFDSM	Only FFDSM	FFDSM + 3D ABUS	Only FFDSM	FFDSM + 3D ABUS	Only FFDSM	FFDSM + 3D ABUS
Skaane et al. [3]	2015	90	54.9	17	13	28	37	11	n.a	18	35	53	n.a.	n.a.	n.a	6.56	n.a	n.a	n.a	n.a	n.a	n.a	n.a
Chang et al. [4]	2011	67	49.5	20	24	46	n.a	8	16	n.a	n.a	n.a	15.5	13.5	−2	n.a	n.a	n.a	n.a	n.a	92.5	n.a	63
Giuliano et al. [5]	2013	3418	54	n.a	n.a	n.a	n.a	n.a	n.a	19	23	42	21.3	14.3	−7	1.25	0.65	12.3	4.6	76	96.7	98.2	99.7
Rella et al. [6]	2018	n.a	n.a	n.a	n.a	n.a	n.a	n.a	n.a	n.a	n.a	0	n.a	n.a	0	n.a	8.3	n.a	n.a	n.a	n.a	n.a	n.a
Kolb et al. [7]	2002	221	50	n.a	n.a	n.a	59	46	146	75	67	142	13.5	14.7	1.2	n.a.	n.a.	2.73	n.a	77.6	75.3	98.8	96.8
Mohammed et al. [8]	2018	25	49.5	9	4	25	5	4	3	17	20	37	n.a	n.a	0	n.a	n.a	100	100	95	85	n.a	n.a
Vaughan et al. [9]	2016	58	50.4	n.a	n.a	n.a	2	1	n.a	7	7	14	n.a	n.a	0	n.a	n.a	n.a	n.a	n.a	n.a	n.a	n.a
Geisel et al. [10]	2018		50	n.a	n.a	n.a	n.a	n.a	n.a	n.a	n.a	0	n.a	n.a	0	n.a	28.4	n.a	n.a	n.a	n.a	n.a	n.a
Wilczek et al. [11]	2016	1668	49	999	699	n.a	n.a	n.a	n.a	7	4	11	22.4	21.8	−0.6	2.2	0.9	6.6	4.2	63.75	100	99	98.4
Giger et al. [12]	2016	185	49	n.a	n.a	n.a	n.a	n.a	n.a	n.a	n.a	0	n.a	n.a	0	n.a	n.a	n.a	n.a	57.5	74.1	78.8	76.2
Vourtsis et al. [13]	2018	1886	48.6	n.a	n.a	97	57	15	22	n.a	n.a.	0	n.a	n.a.	0	n.a.	n.a.	n.a.	n.a.	n.a.	n.a.	n.a.	n.a.
Lee et al. [14]	2019	121	55	n.a	n.a	n.a	1	n.a	n.a	n.a	n.a	0	n.a	n.a	0	8.8	8.3	n.a.	n.a.	n.a.	n.a.	n.a.	n.a.
Elkhalek et al. [15]	2019	25	49	9	4	20	5	4	3	5	7	12	n.a.	n.a.	0	n.a.	n.a.	n.a	n.a	85	100	62.5	100
Kim et al. [16]	2020	38	n.a	n.a	n.a	66	n.a	n.a	n.a	n.a	n.a	0	n.a	n.a	0	n.a	n.a.	n.a	n.a	98	92	62.5	87.5
Park et al. [17]	2018	49	50	n.a	n.a	n.a	n.a	n.a	51	n.a	n.a	0	0.54	0.34	−0.2	n.a	n.a.	n.a	n.a	44.7	22.2	97.6	95.2
Brem et al. [18]	2015	15,318	53.3	n.a	n.a	n.a	11.488	3830	n.a	17	95	112	n.a	n.a	0	28.4	28.4	7.3	5.4	73.2	100	85.4	72
Thigpen et al. [19]	2018	n.a	n.a	n.a	n.a	n.a	n.a	n.a	n.a	n.a	n.a	n.a	n.a	n.a	n.a	n.a	n.a	n.a	n.a	n.a	n.a	n.a	n.a
Mudinger et al. [16]	2016	n.a	n.a	n.a	n.a	n.a	n.a	n.a	n.a	n.a	n.a	n.a	n.a	n.a	n.a	n.a	n.a	n.a	n.a	n.a	n.a	n.a	n.a
Nicosia et al. [20]	2020	n.a	n.a	n.a	n.a	n.a	n.a	n.a	n.a	n.a	n.a	n.a	n.a	n.a	n.a	n.a	n.a	n.a	n.a	n.a	n.a	n.a	n.a
Mengmeng et al. [21]	2020	937	49.1	n.a	n.a	n.a	624	20	34	31	31	62	n.a	n.a	0	4.2	n.a.	n.a.	0.94	n.a.	99.1	n.a.	86.9
Kelly et al. [22]	2010	4419	53	n.a	n.a	n.a	n.a	n.a	n.a	23	46	69	n.a	n.a	0	9.6	9.6	7.2	3.6	40	81	95.1	98.7
Wenkel et al. [23]	2008	35	n.a	n.a	n.a	25	n.a	n.a	n.a	n.a	n.a	n.a	n.a	n.a	n.a	n.a	n.a	n.a	n.a	n.a	n.a	n.a	n.a
Prosch et al. [24]	2011	148	53	n.a	n.a	n.a	9	9	0	n.a.	n.a.	0	n.a.	n.a.	0	n.a.	n.a.	n.a.	n.a.	83.3	81.2	99.1	97.8
Wilczek et al. [25]	2013	1676	53	n.a	n.a	14	n.a	n.a	n.a	6	4	10	25	40	15	n.a	n.a.	n.a	n.a	n.a	n.a	n.a	n.a
Gatta et al. [26]	2021	1165	50	729	436	n.a	40.3	32.22	26	4	8	12	16.6	16.96	0.36	31	−14	3.4	6.8	58.8	93.5	94	87
		31.549	50.86	1180	1180	3.21	12.327	39.802	301	229	347	576	11.484	121.6	6.76	91.51	−12.45	13.953	12.554	8527	1100	9703	11.592

**Table 2:** The table shows the publications included and the evaluations from two independent readers and, if necessary, from a third reader.

Patientes Data						Lesions	BI RADS			Cancer Found			Size Cancer mm		Recall Rate (‰)			Cancer Detection Rate (‰)		SE (%)		SP (%)	
Autors	Y	N. Patients	Age	ACR3	ACR4		3	4	5	ONLY FFDSM	FFDSM + 3D ABUS	TOT	ONLY FFDSM	FFDSM + 3D ABUS	ONLY FFDSM	FFDSM + 3D ABUS	DIFF	ONLY FFDSM	FFDSM + 3D ABUS	ONLY FFDSM	FFDSM + 3D ABUS	ONLY FFDSM	FFDSM + 3D ABUS
Skaane et al. [3]	2015	90	54.9	17	13	28	37	11	n.a.	18	35	53	n.a.	n.a.	n.a	6.56	n.a	n.a	n.a	n.a	n.a	n.a	n.a
Giuliano et al. [5]	2013	3418	54	n.a	n.a	n.a	n.a	n.a	n.a	19	23	42	21.3	14.3	−7	1.25	0.65	12.3	4.6	76	96.7	98.2	99.7
Kolb et al. [7]	2002	221	50	n.a	n.a	n.a	59	46	146	75	67	142	13.5	14.7	1.2	n.a.	n.a.	2.73	n.a	77.6	75.3	98.8	96.8
Mohammed et al. [8]	2018	25	49.5	9	4	25	5	4	3	17	20	37	n.a	n.a	0	n.a	n.a	100	100	95	85	n.a	n.a
Wilczek et al. [11]	2016	1668	49	999	699	n.a	n.a	n.a	n.a	7	4	11	22.4	21.8	−0.6	2.2	0.9	6.6	4.2	63.75	100	99	98.4
Giger et al. [12]	2016	185	49	n.a	n.a	n.a	n.a	n.a	n.a	n.a	n.a	0	n.a	n.a	0	n.a	n.a	n.a	n.a	57.5	74.1	78.8	76.2
Park et al. [17]	2018	49	50	n.a	n.a	n.a	n.a	n.a	51	n.a	n.a	0	0.54	0.34	−0.2	n.a	n.a.	n.a	n.a	44.7	22.2	97.6	95.2
Brem et al. [18]	2015	15.318	53.3	n.a	n.a	n.a	11.488	3830	n.a.	17	95	112	n.a	n.a	0	28.4	28.4	7.3	5.4	73.2	100	85.4	72
Mengmeng et al. [21]	2020	937	49.1	n.a	n.a	n.a	624	20	34	31	31	62	n.a	n.a	0	4.2	n.a.	n.a.	0.94	n.a.	99.1	n.a.	86.9
Kelly et al. [22]	2010	4419	53	n.a	n.a	n.a	n.a	n.a	n.a	23	46	69	n.a	n.a	0	9.6	9.6	7.2	3.6	40	81	95.1	98.7
Gatta et al. [26]	2021	1165	50	729	436	n.a	40.3	32.22	26	4	8	12	16.6	16.96	0.36	31	−14	3.4	6.8	58.8	93.5	94	87
		12.192	355	1754	1152	53	736.49	3911	260	211	329	540	74.34	68.1	6.24	83.21	−12.45	125.54	139.5	586.4	826.9	746.2	810.9

## Data Availability

No new data were created or analyzed in this study. Data sharing is not applicable to this article.
